# ^1^H-NMR-Based Metabolomics: An Integrated Approach for the Detection of the Adulteration in Chicken, Chevon, Beef and Donkey Meat

**DOI:** 10.3390/molecules26154643

**Published:** 2021-07-30

**Authors:** Muhammad Tayyab Akhtar, Muneeba Samar, Anam Amin Shami, Muhammad Waseem Mumtaz, Hamid Mukhtar, Amna Tahir, Syed Shahzad-ul-Hussan, Safee Ullah Chaudhary, Ubedullah Kaka

**Affiliations:** 1 Institute of Industrial Biotechnology, GC University, Lahore 54000, Pakistan; muneebasamar@live.co.uk (M.S.); anamshami61@gmail.com (A.A.S.); hamidmukhtar@gcu.edu.pk (H.M.); 2 Department of Chemistry, University of Gujrat, Gujrat 50700, Pakistan; muhammad.waseem@uog.edu.pk; 3 Syed Babar Ali School of Science and Engineering, Lahore University of Management and Sciences, Lahore 54792, Pakistan; amnatahir80@gmail.com (A.T.); shahzad.hussan@lums.edu.pk (S.S.-u.-H.); safeeullah@lums.edu.pk (S.U.C.); 4 Department of Companion Animal Medicine and Surgery, Faculty of Veterinary Medicine, University Putra Malaysia, UPM, Serdang 43400, Selangor, Malaysia

**Keywords:** metabolomics, NMR, multivariate data analysis, biomarkers, halal meat

## Abstract

Meat is a rich source of energy that provides high-value animal protein, fats, vitamins, minerals and trace amounts of carbohydrates. Globally, different types of meats are consumed to fulfill nutritional requirements. However, the increasing burden on the livestock industry has triggered the mixing of high-price meat species with low-quality/-price meat. This work aimed to differentiate different meat samples on the basis of metabolites. The metabolic difference between various meat samples was investigated through Nuclear Magnetic Resonance spectroscopy coupled with multivariate data analysis approaches like principal component analysis (PCA) and orthogonal partial least square-discriminant analysis (OPLS-DA). In total, 37 metabolites were identified in the gluteal muscle tissues of cow, goat, donkey and chicken using ^1^H-NMR spectroscopy. PCA was found unable to completely differentiate between meat types, whereas OPLS-DA showed an apparent separation and successfully differentiated samples from all four types of meat. Lactate, creatine, choline, acetate, leucine, isoleucine, valine, formate, carnitine, glutamate, 3-hydroxybutyrate and α-mannose were found as the major discriminating metabolites between white (chicken) and red meat (chevon, beef and donkey). However, inosine, lactate, uracil, carnosine, format, pyruvate, carnitine, creatine and acetate were found responsible for differentiating chevon, beef and donkey meat. The relative quantification of differentiating metabolites was performed using one-way ANOVA and Tukey test. Our results showed that NMR-based metabolomics is a powerful tool for the identification of novel signatures (potential biomarkers) to characterize meats from different sources and could potentially be used for quality control purposes in order to differentiate different meat types.

## 1. Introduction

Meat is one of the most consumed foods around the world [1]. The importance of meat as a dietary source could be established from its rich nutritional contents which includes proteins, good fats like omega-3-fatty acids, and the abundant presence of vitamins like B6 and B12 and zinc [1,2]. However, in the case of fats, their content varies according to the type of meat and the amount of meat consumed by the individual [3].

High economic value and increased demand for meat have burdened the meat industry, which leads to the substitution of high-quality meat with low quality [4]. The situation has also triggered the deliberate adulteration of high-priced meat species with cheaper ones. Identification of meats of various food species is of utmost importance because of social, forensic and public health reasons. The adulterated meat often enters the supply chain and jeopardizes the sentiments as well as the health of the people. A person may have an allergy or other medical issues with eating a particular type of meat; therefore, it is mandatory or morally/legally obligatory to properly label the meat type in order to avoid health issues. Recently, beef meatballs were found to be adulterated with wild boar meat [5], while some of the European marketed food items were reported to have undeclared horse meat [6]. Similarly, in the United Kingdom a number of meat pies labeled as halal were found to have pork DNA [7]. Furthermore, the followers of Islam and Judaism have some religious concern regarding the consumption of non-halal meat, which would cause a decline in the consumption of meat products and affect their sentiments [8]. Therefore, pinpointing the meat source from different food animals is an important issue regarding social and forensic concerns [9]. Thus, keeping in view of the current alarming situation, it is of immense importance to use advanced analytical techniques, which could facilitate to differentiate between meat samples from various species and sold in the market as a substitute of the others, which normally are not demanded or would not be healthy from public health concern.

Metabolomics is an emerging field and a powerful tool for discovering biomarker signatures, particularly for early disease detection, and also contributing to assessing the quality and safety of food and raw food items [10]. Metabolomics focuses on the high-throughput characterization of small molecules (metabolites) in biological matrices and provides a broader view of changes occurring in metabolites over time [11]. The qualitative and quantitative analysis of these metabolites is performed through different analytical techniques such as Nuclear magnetic resonance (NMR) and mass spectrometry (MS) [12].

Nuclear Magnetic Resonance (NMR) is a universal, qualitative and quantitative analytical technique used to identify and quantify chemicals from complex biological mixtures. The unbiased view of the sample composition, simple sample preparation, non-destructive sampling, short analysis time and simultaneous quantitation of multiple compounds are the features that make NMR the method of choice for metabolomics studies. NMR, along with multivariate data analysis (MvDA) (e.g., Principal component or Partial least square discriminant analysis), is a power full tool that generates a wealth of data that can be processed to identify important biomarkers (bio-chemicals/metabolites) that can help to solve many biological puzzles. Previously, ^1^H-NMR spectroscopy was used to differentiate beef from horse meat, and triglyceride ratio was identified as a major discriminating factor [13]. A similar approach has been used to perform metabolite profiling of different beef secretions [14] and also differentiation of irradiated and non-irradiated ground beef [15]. Likewise, Xiao et al. [16] studied chemical composition of the forerunner flavor substance of Wuding chicken at different ages by using ^1^H-NMR spectroscopy.

Additionally, Bischof et al. [17] used ^1^H-NMR spectroscopy technique in order to analyze the effect of dry and wet aging on the beef metabolome. Another study was conducted using an untargeted NMR metabolomic approach to evaluate the raw meat metabolome including the metabolic differences between multiple muscle specimens of locally farmed Jiulong yaks [18]. A similar platform was used to study the effect of dark cutting meat on meat quality attributes and concentrations of post-mortem glycolytic metabolites in Angus x Nellore cross breed cattle [19]. Two-dimensional quantitative NMR spectroscopy in combination with MvDA was also proved to be an efficient method to distinguish the breast meat of four native chicken strains from broiler breast meat [20]. Muroya et al. [21] discussed in detail the significance of NMR along with MvDA as a practical tool to monitor meat quality traits effectively.

The previous studies were more concerned to investigate the effect of various treatments, e.g., forerunner flavor substance [16], dry and wet aging [17] or dark-cutting meat [19] on the metabolome of animal’s meat. Likewise, earlier reports also highlighted the potential biomarkers related to meat quality traits with a focus on specific aspects such as animal genetic background [22], sensory attributes [23], feeding system [22] and formulations [24], including processes such as postmortem storage [25] and hygiene control. [21]. Additionally, most of the literature revealed the discriminatory analysis of multiple muscle specimens belonging to the same species. Here, we have used ^1^H-NMR based metabolomics to differentiate various meat types belong to different species. The aim of the current study is to address the issue of meat adulteration, particularly in the regions where low-priced meat e.g., donkey meat, is mixed with high-priced meat like beef and chevon. Moreover, the present data also exhibited metabolites responsible for differentiating white meat (chicken) from red meat.

## 2. Results

### 2.1. ^1^H-NMR Identification of Metabolites in Different Meat Samples

**Figure 1:**
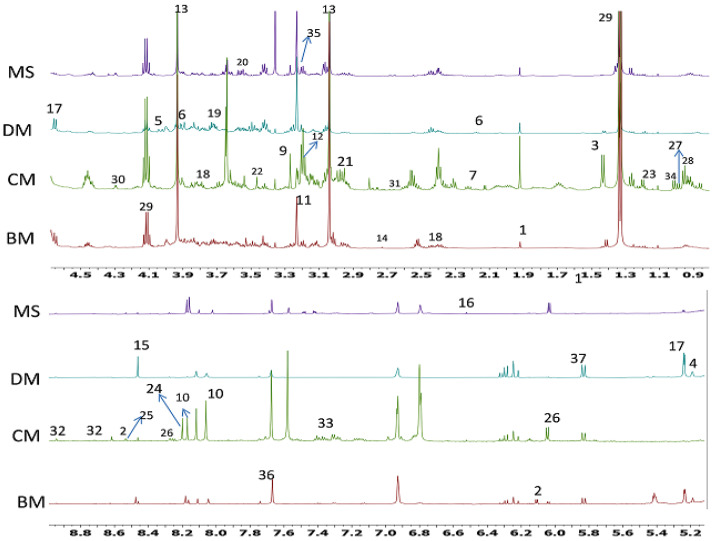
H-NMR spectra of MS (Chevon); DM (Donkey); BM (Beef); CM (Chicken). 1, Acetate; 2, ADP/AMP/ATP; 3, alanine; 4, α-Mannose; 5, Asparagine; 6, Aspartic acid; 7, Biotin; 8, Butyric acid; 9, betaine; 10, carnosine; 11, carnitine; 12, choline; 13, creatine; 14, dimethylamine; 15, formate, 16, fumarate; 17, glucose; 18, glutamate; 19, glutamine; 20, glycerol; 21, glutathione; 22, glycine; 23, 3-hydroxybutyrate; 24, hypoxanthine; 25, IMP; 26, inosine; 27, isoleucine; 28, leucine; 29, lactate; 30, malate; 31, methionine; 32, niacinamide; 33, phenyalanine; 34, valine; 35, phosphocholine; 36, pyruvate; 37, uracil.

The comparison between representative ^1^H-NMR spectra of Chevon (MS), Chicken (CM), Beef (BM) and Donkey meat (DM) is shown in Figure 1. The stacked ^1^H-NMR spectra of all the biological replicates of each meat type are shown in Supplementary Figure S1. Visual inspection of Figure 1 shows that all the meat groups had similar peaks in ^1^H-NMR spectra; however, the higher signal intensities in CM indicated a higher level of metabolites in the Chicken group. In Figure 1, spectra shown from all four meat types were stacked together; some of the identified signals were not seen in a few meat types, which might be due to the higher intensity of signals in CM. In total, 37 metabolites were identified from all four meat types. The list of identified metabolites is shown in Table 1. The identified metabolites included amino acids (alanine, asparagine, aspartic acid, glutamine, isoleucine, leucine, and valine), dipeptide (carnosine), organic acids (3-hydroxybutyrate, acetate, creatine, formic acid), vitamins (biotin, glutathione), sugars (glucose, glycerol and α-mannose), nucleosides (inosine, hypoxanthine, uracil), and energy related metabolites e.g., lactate, carnitine, or ADP/AMP/ATP. Overall, stacked ^1^H-NMR spectra were dominated by signals of creatine and lactate.

### 2.2. Multivariate Data Analysis

**Figure 2:**
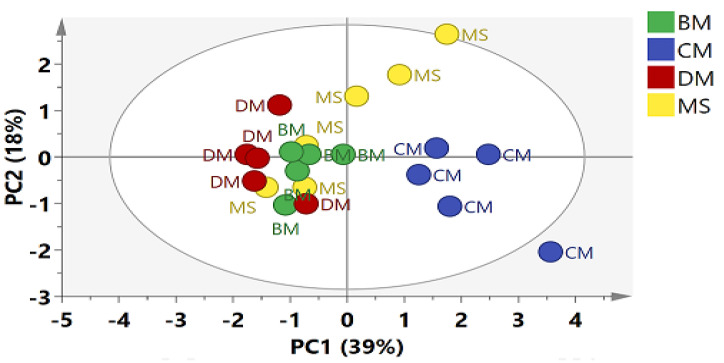
Score plot (PC1 vs. PC2) of PCA based on the whole range of ^1^H-NMR signals (δ 0.5–δ 10.0) of CM = Chicken; MS = Chevon; BM = Beef; DM = Donkey.

**Figure 3:**
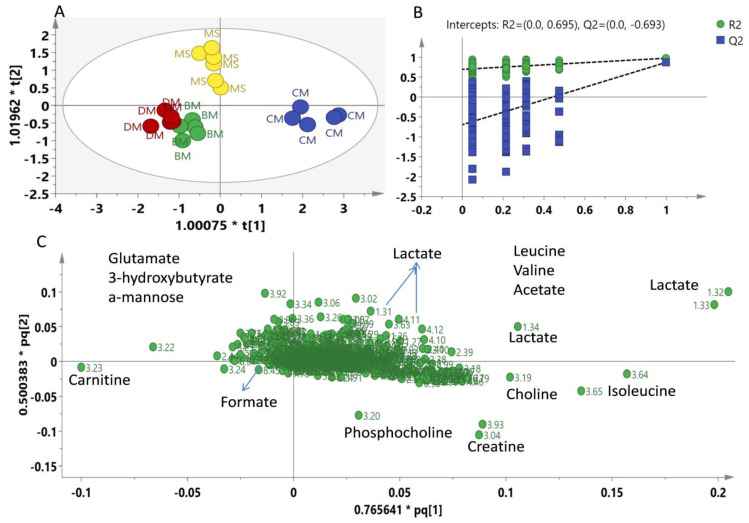
(**A**) Score plot (PLS1 vs. PLS2) of OPLS-DA based on whole range of1^1^H-NMR signals (δ 0.5–δ 10.0) of CM = Chicken; MS = Chevon; BM = Beef; DM = Donkey. (**B**) Validation through 200 permutation test, (**C**) OPLS-DA Loading scatter plot.

The processed ^1^H-NMR data were subjected to principal component analysis (PCA) in order to observe the similarities and differences among the meat types. In PCA, five components were able to explain 83% of the variation. PC1 contributed 39%, whereas PC2 was able to explain 18% variation present in the data (Figure 2). PCA score plot showed a separation among CM and the rest of the groups. BM and DM were clustered together on the negative side of PC1, whereas half of the MS samples were overlapped with BM and DM, and half were projected close to the CM group with a positive PC1 score. Altogether, PCA was not able to differentiate all four meat groups. Later, orthogonal partial least square-discriminant analysis (OPLS-DA) was applied to the same data. The OPLS-DA score plot showed a clear differentiation among meat types (Figure 3A). The model showed high goodness of fit and fairly good predictability, with R^2^Y equal to 0.86 and Q^2^ equal to 0.63. The model was validated through a 200 permutation test and Q^2^ of value −0.693 was found, as shown in Figure 3B. The OPLS-DA score plot showed a clear separation between white meat (CM) and red meat (MS, BM, DM). The CM group was separated from the rest of the meat groups and clustered on the positive side of PLS1, whereas the other three meat groups belonged to red meat, which were projected to the negative side of PLS1. However, MS was different from BM and DM on the basis of PLS2 with a positive PLS2 score, while BM and DM were clustered together on the lower left quadrant, having a negative PLS2 score. The corresponding loading plot shows the metabolites responsible for the separation of meat groups in the score plot (Figure 3C). The signals of lactate, creatine, phosphocholine, choline, leucine, isoleucine, valine and acetate were found on the positive side of PSL1 corresponded to the CM group, whereas Glutamate, 3-hydroxybutyrate and α-mannose were present in higher level in the MS group while carnitine and formate were found to be responsible for the separation of BM and DM, placed on lower left quadrant of loading scatter plot (Figure 3C). The loading column plot of PLS1 is shown in Supplementary Figure S2.

**Figure 4:**
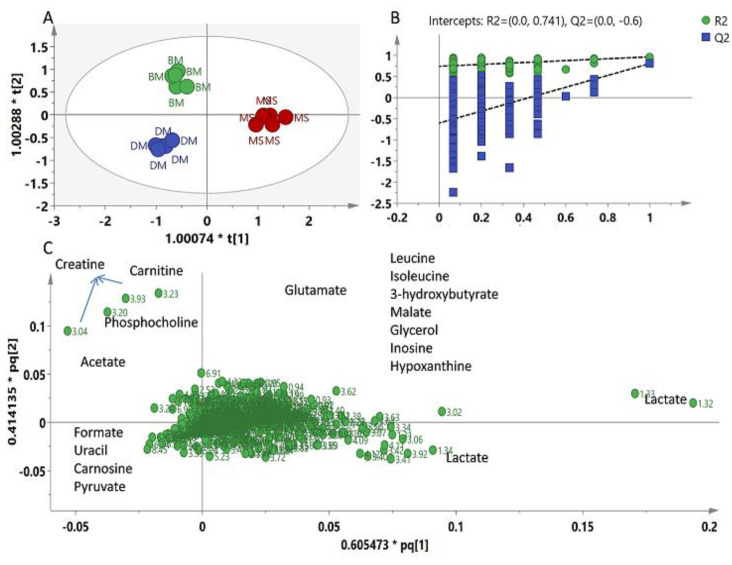
(**A**) Score plot (PLS1 vs. PLS2) of OPLS-DA based on whole range of ^1^H-NMR signals (δ 0.5–δ 10.0) of MS = Chevon; BM = Beef; DM = Donkey. (**B**) Validation through 200 permutation test. (**C**) Loading scatter plot of OPLS-DA.

Although component 3 also showed separation between BM and DM (data shown in Supplementary Figure S3), we excluded the CM group from the analysis, and the rest of the data were subjected to OPLS-DA in order to study the difference between red meat types. The model showed high goodness of fit with high predictability and with R^2^Y equal to 0.97 and Q^2^ 0.72, respectively. A tight clustering among the samples of all three meat types and a clear separation between meat groups can be seen in the score plot (Figure 4A). Like in the previous model, MS was clearly differentiated from BM and DM found on the positive side of PLS1, while BM and DM were observed on the negative side of PLS1 but well separated on the base of PLS2 projecting to positive and negative PLS2 quadrants, respectively. The model was validated through a 200 permutation test and Q^2^ value −0.6 was found as shown in Figure 3B. Along with the number of metabolites shown in the loading plot, lactate was the major compound responsible for the separation of MS from BM and DM (Figure 4C). Moreover, carnitine and creatine were found corresponding to BM, while formate, carnosine, uracil and pyruvate were found to be the major differentiating metabolites corresponding to DM. Due to a higher number of variables (chemical shifts), it was difficult to show the chemical shifts of all the identified metabolites in the loading scatter plot; therefore, some of the metabolites listed in the loading plot were identified after “zooming in” the loading plot in Simca software. However, for the convenience of the reader, important loading column plots have been added in Supplementary Figure S4.

### 2.3. Relative Quantification

**Figure 5:**
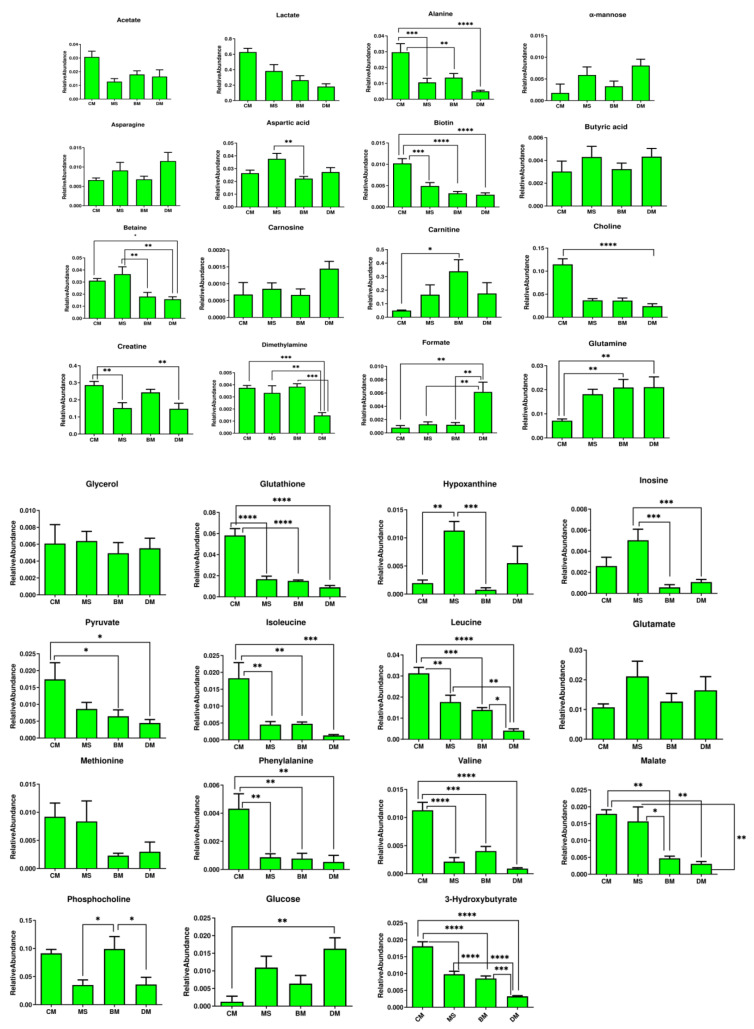
Relative quantification of the major differentiating metabolites based on the mean peak area of the related signals. CM = Chicken; MS = Chevon; BM = Beef; DM = Donkey. * depicts the differences between meat groups. Statistical differences of metabolites between groups were calculated by using one-way analysis of variance and a Tukey multiple comparison test. Statistical icons: * *p* < 0.05, ** *p* < 0.01, *** *p* < 0.001, and **** *p* < 0.0001.

The relative quantification of the differentiating metabolites was done by using one-way ANOVA and Tukey test (for multiple comparisons), as shown in Figure 5. The relative quantification table and graph also showed the comparative level of differentiating metabolites (between white and red meat and also among the red meats) in all the studied meat types (Supplementary Table S1).

## 3. Discussion

Nuclear Magnetic Resonance (NMR) is a universal, qualitative and quantitative analytical technique. It is a powerful method that can interpret the structure, dynamics and biological interaction of macromolecules. NMR spectroscopy can be used to identify and quantify chemicals from complex mixtures. An unbiased view of the sample composition and simultaneous quantification of multiple compounds are the features that make NMR the method of choice for metabolomics studies [26]. It is a non-destructive and reliable technique that does not require greater sample preparation time and is fast in working. Hence, NMR can be used to make databases in situations where a lot of samples are to be analyzed in a diminutive time [22]. Targeted metabolite profiling or quantitative metabolomics helps in identifying and quantifying multiple compounds. This can be done by comparing the mixture of chemical compounds present in the NMR data sample to a spectral library of reference derived from pure compounds of known concentrations. When these certain compounds are identified, they are statistically analyzed to identify essential biomarkers. Quantitative metabolomics can be selective; that is, they can approach a specific class of compounds such as amino acids or nucleic acids, or it can be widespread to all detectable compounds [27].

There are many reports on using NMR spectroscopy along with MvDA to perform meat characterization, e.g., to study the geographical origin of meat, for differentiation among various cattle breeds together with identification of healthy or diseased animal meat, quality control and to study the effects of various treatments (i.e., effects of different feeds) on meat quality. Previously, along with muscle tissues, ^1^H-NMR spectroscopy has been used vigorously to carry out the metabolite profiling of urine, liver, serum and plasma samples obtained from various animals [28,29]. The Apulian lamb meat samples collected from three different locations in southern Italy were differentiated on the basis of their geographical origin by using NMR spectroscopy [30].

The level of some of the metabolites seems to play a very important role in order to differentiate meat of different species or even the breeds within the same species. For example, the level of lactate was reported to be a decisive factor in differentiating different cross-breeds of pig [31]. In another study, lactate along with other metabolites was found to be affected by the cross breeds of pig, whereas carnosine was correlated to the low value of sensory traces related to the flavor of the meat [23]. In the current study, lactate was not only found to differentiate chicken from chevon, beef and donkey meat, but it was also observed as a major differentiating metabolite to separate chevon from beef and donkey meat. In cured meat, the presence of lactic acid bacteria is desirable compared to other aerobic spoilage bacterial strains; it ensures maximum shelf-life, safety and stability of fermented meat [32]. Moreover, the higher level of carnosine in donkey meat might also indicate lower sensory traits of donkey meat than beef and chevon.

Lactic acid is an immediate source of energy under stress conditions. The amount of lactic acid produced depends upon the stress condition prior to postmortem. Therefore, in stressed muscle, an excess of lactic acid will be generated to keep balance in ATP content right after slaughter [33]. Furthermore, the concentration of lactic acid produced in the body depends on the type of muscle and its glycogen content [34]. Oxidative muscle utilizes an ample amount of lactate, which serves as an eminent source of energy [35]. Lactic acid is continuously produced in the body and converted back to pyruvate under normal conditions. However, after slaughter, the flow of lactate ions from muscles to interstitial fluid becomes slow, thus maintaining postmortem homeostasis. Therefore, excess of lactic acid will decrease pH, which will ultimately affect meat quality [33]. In the present study, the amount of lactic acid produced in red meat is comparatively high as compared to white meat, so it is an attribute for differentiating among meat samples. Furthermore, donkey meat has a significantly high concentration of lactic acid in comparison to beef and chevon. This implies that high postmortem lactic acid production in donkey meat would affect its quality and palatability. The onset of rigor mortis causes a decline in ATP, which degrades immediately into ADP, AMP and eventually IMP. IMP is further depleted into inosine and hypoxanthine [36]. Inosine serves as a biomarker in determining meat quality [37]. Additionally, previous data showed that inosine protects mice against γ-radiation-induced death by reducing the production of ROS [38]. In our study, we found that inosine is responsible for differentiating between donkey, chevon and beef meat samples. It is substantially present in high concentrations in beef and donkey, which would affect its flavor and quality.

Amino acids are the key compounds in growth and immunity, as well as in regulating metabolic pathways [39]. Concomitantly, they play a crucial role in developing meat flavor [40]. Amino acids like alanine, glycine and serine impart a sweet taste while histidine, arginine, isoleucine and leucine impart a sour taste [41]. Branched-chain amino acids (BCAAs) like leucine, isoleucine and valine serve as signaling molecules in various metabolic pathways such as mammalian target of rapamycin (mTOR) [42]. BCAA plays a substantial role in regulating lipolysis, thus ameliorating glucose consumption and improving meat quality [43]. A previous work conducted on finishing pigs reported that leucine supplementation could improve pork texture meliorating protein content and enhancing meat quality [44]. BCCAs are also responsible for improving glucose uptake and utilization by upregulating glucose transporters, thus promoting muscle growth [45]. In the current study, high levels of BCAAs are present in red meat, contrary to white meat. Therefore, it suggests that BCAA would be responsible for enhancing proteinaceous content of red meat, thus claiming it as a potent source of nutritional amino acid. Additionally, a high BCAA content would influence meat quality.

Choline is an indispensable micro-nutrient. It has prominent role in cell membrane integrity and muscle function, as well as in the synthesis of neurotransmitters [46]. Choline serves as a precursor for methyl metabolism, which is involved in creatine biosynthesis [47]. Previous interventions showed that a diet deficient in choline causes serious health problems in humans and animals [48]. Choline can be used in the treatment of fatty liver syndrome [49]. Besides having a dietary role, choline supplementation could improve meat quality. Li et al. [50] studied the impact of rumen-protected choline (RPC) on the longissimus dorsi muscle of lambs. They inferred that supplying lamb with RPC resulted in high pH values post mortem, while low shear force, which influences meat quality. The low pH values are mainly due to the accumulation of lactic acid. High pH values of lamb muscles supplemented with 0.25% RPC might be due to a decline in the production of lactic acid. Therefore, a high choline level would be beneficial both for animals and human. Interestingly, in current metabolomics study, the amount of choline was high in red meat samples (BM, DM, MS) as compared to white meat (CM). As we know that choline is part of a membrane, a high choline level in the red meat samples under study might be related to membrane properties, influencing meat quality. Furthermore, red meat would serve as a source of dietary choline for humans.

Creatine, an amino acid derivative, plays a key role in energy metabolism by maintaining ATP level and hampering the generation of ADP, which would ultimately lead to the formation of reactive oxygen species [51]. Creatine assimilates in the body through intake of meat and meat products, and it escalates muscle performance [52]. A previous study showed that supplementation of creatine to pigs would change postmortem metabolism and affect meat quality [53]. Likewise, Li et al. [54] studied the relation between creatine supplementation on meat quality and postmortem metabolism. He inferred that pigs supplemented with creatine prior to slaughter showed a decline in lactic acid concentration. Therefore, creatine slows down the decrease in postmortem pH levels by maintaining phosphate in muscle cells [47].

In the current work, we observed that creatine is responsible for discriminating between meat samples. Moreover, in contrast to other meat groups, the concentration of creatine was significantly high in chevon and donkey. These findings suggest that an elevated level of creatine would prevent the change in pH due to postmortem lactate production, thus improving meat quality. Carnosine, a naturally occurring dipeptide, is found abundantly in skeletal muscles. It acts as a pH buffer in muscles, a neurotransmitter, an antioxidant and an inhibitor for the end products of glycation and lipoxidation [55]. Carnosine is also involved in reducing the amount of free radicals by directly interacting with ROS [56]. Besides having a nutritive role, carnosine contributes to meat quality. A previous study conducted by Hu et al. [57] assessed the dietary role of carnosine on meat quality, growth performance and oxidative stability. They inferred from their findings that supplementation of carnosine influences both chicken meat quality and quantity. Similarly, Cong et al. [58] demonstrated that dietary supplementation of carnosine resulted in high pH level, redness and cohesiveness of broiler muscle, thus enhancing meat quality. Moreover, carnosine augmentation has enhanced the activities of antioxidant enzymes. Carnosine was also reported to be associated with umami flavor and meat tenderness [59]. In the present study, we observed that carnosine is one of the key metabolites that discriminate between red meat samples (MS, BM and DM). Therefore, a high amount of carnosine would impact meat tenderness and other meat quality traits. Carnitine also provides cellular energy by transporting long-chain fatty acids across the mitochondrial membrane [60]. In our work, we have observed a statistically significant increase in the concentration of carnitine in beef as compared to other meat samples. Moreover, it also takes part in differentiating between chevon, beef and donkey. Therefore, it would serve as a source of energy in different meat types, particularly in beef. In the present study, we have identified several energy-related metabolites like carnosine, carnitine and BCAA, which not only add nutritional value to meat but also contribute to sensory attributes and quality attributes of meat.

Although we have identified 37 metabolites in four meat groups, not all of them contributed to discriminating the meat types. A literature survey and current data showed that in the case of meat, there are some specific metabolites that contribute more than others in meat characterization. Previously, alanine, carnitine, creatine, glutamine, succinate, acetate, betaine, creatinine, glycerol and glycine were found responsible for differentiating Korean and Australian beef samples, whereas betaine, carnosine, creatine, glycerol, glycine, leucine, isoleucine and valine separated New Zealand beef from United States beef [61]. Similarly, valine, leucine, isoleucine, glutamate, glutamine, carnosine, lysine, arginine, acetate, pyruvate, carnitine and taurine were involved in discriminating four cattle breeds [62]. Kim et al. [63] identified glutamate, isoleucine, leucine, tryptophan, phenylalanine, valine and tyrosine as major metabolites for separating dry-aged beef from wet-aged beef samples. Likewise, Kodani et al. [64] reported acetic acid, alanine, glutamic acid, isoleucine, leucine, phenylalanine, tyrosine, valine, carnitine, carnosine, creatine and lactic acid to be important metabolites to discriminate Japanese black cattle on the basis of their ages. Interestingly, in almost all the above-mentioned reports, OPLS-DA was admired as a good choice for discriminating meat samples. Consistent with previous reports, we have also found OPLS-DA as a good choice for differentiating meat groups and regarding metabolites, lactate, creatine, choline, acetate, leucine, isoleucine, valine, formate, carnitine, glutamate, 3-hydroxybutyrate and α-mannose, which have already been reported as important biomarkers in meat characterization, were found as the major discriminating metabolites between white (chicken) and red meat (chevon, beef and donkey). Meanwhile, inosine, lactate, uracil, carnosine, formate, pyruvate, carnitine, creatine and acetate were responsible for differentiating chevon, beef and donkey meat.

NMR-based metabolomics has also been successfully applied to white meat to study the effects of breed [65], age [66,67], pH [68], diet [69] and different treatments (i.e., boiling processes) [70] on meat quality as well as the cause of infections, i.e., pectoralis muscle dystrophy [71]. Contrary to previous data, our results depict a clear metabolic difference between white and red meat and also differentiate the meat types that belong to red meat. We have found NMR-based metabolomics to be a powerful tool to differentiate different meat groups and can be used as a good analytical tool in the meat industry to address the issue of meat adulteration. The discriminating metabolites identified in the result of MvDA can be used as potential biomarkers for quality control purposes, particularly in countries facing the problem of illegal supply/marketing of meat types that are strictly banned to consume either by religious law or under the jurisdiction of the country.

## 4. Materials and Methods

### 4.1. Chemicals

The chemicals used in this research were 6% perchloric acid (HClO_4_), potassium carbonate (K_2_CO_3_) and liquid nitrogen. Deuterium oxide (D_2_O) (Cat-No: 14D-099) and sodium-3-trimethylsilylpropanoic acid (TMSP or TSP) (Cat-No: I-18625) were obtained from Cambridge Isotope Laboratories (Andover, MA 01810, USA).

### 4.2. Biological Samples Collection

For sample collection, one cut of fresh meat (gluteal muscle) weighing approx. 1 g was taken from six goats and six cows from Punjab Agriculture and Meat Company (PAMCO) slaughter house near Shahpur Kanjraan, Lahore. Similarly, six different chicken meat samples (gluteal muscle) weighing approx. One gram of each was collected from a local chicken vendor in Lahore. The six donkey meat samples were collected from the University of Veterinary and Animal Sciences (UVAS) Pattoki campus. For the classification purpose, samples from goat, beef, chicken and donkey were labeled as chevon sample (MS), beef meat (BM), chicken meat (CM) and donkey meat (DM), respectively. All the samples were collected in sterile tubes containing liquid nitrogen and transported in an ice box. After the collection, all 24 properly labeled samples were stored at −8 °C until further processing. The animal protocol was approved by the animal care committee of Government College University, Lahore.

### 4.3. Sample Preparation for NMR Spectroscopy

Meat samples were prepared for NMR analysis according to established protocol [29]. All frozen meat samples were pulverized with pestle and mortar into fine powder with the help of liquid nitrogen. Then, from each sample, 0.12 g (120 mg) pulverized meat was weighed and kept in sterile eppendorf. Later, 0.5 mL (500 µL) of ice-cold 6% perchloric acid was added in each eppendorf containing powdered tissue and was vortexed for 30 s. After vortex, samples were incubated on ice for 10 min and then centrifuged for 15 min at 12,000 rpm and the temperature kept constant at 4 °C. Supernatant was obtained and pellets were discarded. For the adjustment of pH of solution, K_2_CO_3_ was used and pH was kept in between 7–8. All these neutralized samples were again incubated on ice for 30 min. After ice incubation, samples were again centrifuged at 12,000 rpm at 4 °C for the removal of KClO_4_ (potassium perchlorate) precipitates. The resulting supernatant of samples obtained was lyophilized into fine powder and stored at −80 °C until NMR analysis.

### 4.4. NMR Spectroscopy

For acquisition of NMR spectroscopy, all the lyophilized samples were dissolved in 500 μL D_2_O, with 0.01% Trimethylsilylpropanoic (TSP) used as a reference compound. The pH of all the solutions was 7.4, and the final volume was 550 μL.

^1^H-NMR data acquisition was done at 600 MHz (Avance neo) NMR spectrometer. The experiments were run with 128 scans and 64 K data points using Bruker’s pulse program “ZG” without solvent suppression. Line broadening (lb = 0.3) was used while processing; however, no zero filling was added.

### 4.5. NMR Spectral Data Analysis

Each spectrum obtained was analyzed by using MestReNova (14.1.2) software (Escondido, CA, USA). Phase and base line correction was done manually. Internal standard (TSP) was used as chemical shift reference and set at 0.0 ppm. This internal standard shift defines all chemical shifts for metabolites and helps in the identification. The intensities of ^1^H-NMR spectra were scaled to total intensity and reduced to integrated regions of equal width (0.01 ppm) corresponding to the region of δ 0.5–δ 10.0. Later, normalization of NMR spectra was done to the sum of all integral regions, in total, 950 variables were obtained which subjected to MvDA. Spectral libraries such as Human Metabolome Database (HMDB) and previously published literature were used for metabolite identification. One-way ANOVA and Tukey test was used for the metabolite quantification using Graph Pad Prism (8.4.3) software (San Diego, CA, USA).

After spectral processing, the NMR data were subjected to MvDA by using SIMCA (Umetrics, Version 14.1). In total, 24 NMR spectra were subjected to principal component analysis (PCA) and partial least square analysis (PLS). The regions of δ 4.8–δ 4.9 were excluded from the analysis because of the residual signal of the deuterated solvent. PCA was performed with the SIMCA-P software based on a Pareto scaling method. Pareto scaling provides a better weight to the variables of the data with superior intensity. It is conducted for a big dynamic range in the data set [59]. The data were further subjected to orthogonal partial least square-discriminant analysis (OPLS-DA).

### 4.6. Statistical Analysis

The differentiating metabolites obtained from MvDA were relatively quantified by using Graphpad prism software (8.44 version). A one-way ANOVA was performed to check a significant level of differences in meat groups on the basis of metabolites, while Tukey’s test of multiple comparision was conducted to evaluate paired differences between the means of metabolites [59]. The statistical analysis was performed with a 95% confidence level, and a probablistic value (*p* < 0.05) indicates statistical significance. Further, descriptive statistics was applied to calculate the standard error mean of each metabolite using Graphpad prism (8.44 version).

## 5. Conclusions

Here, we have used ^1^H-NMR spectroscopy along with MvDA to differentiate various meat types. The spectral data acquired from NMR provided useful information, and a total of 37 metabolites were identified in chicken, chevon, beef and donkey meat. In MvDA, models developed were successfully validated and were able to discriminate all the meat groups. The discriminating metabolites can be used as potential biomarkers to differentiate meats that are difficult to characterize on the basis of visual inspection. Our results verified ^1^H-NMR-based metabolomics as a robust technique to be used in the meat industry for quality control. Moreover, the current study can also be useful for the countries facing the problem of meat adulteration.

## Figures and Tables

**Table 1:** Metabolites identified in different meat samples.

Number	Metabolites	Multiplicity
1	Acetate	1.92 (s)
2	ADP/AMP/ATP	6.11(d), 8.27(s); 8.54(s)
3	Alanine	1.48/1.43 (d)
4	α -Mannose	5.18 (d)
5	Asparagine	2.94 (m), 4.00 (dd)
6	Aspartic acid	3.90 (dd)
7	Biotin	2.22 (t), 3.10 (m)
8	Butyric acid	0.860 (t); 2.175 (t)
9	Betaine	3.27 (s), 3.90 (s)
10	Carnosine	8.18 (s), 8.06 (s), 7.12 (s), 2.7 (m), 4.5 (m), 2.97 (dd)
11	Carnitine	2.40 (m), 3.23 (s)
12	Choline	3.18 (s), 4.06 (m)
13	Creatine	3.04 (s), 3.93 (s)
14	Dimethylamine	2.73 (s)
15	Formate	8.45 (s)
16	fumarate	6.51 (s)
17	Glucose	4.64 (d), 5.24 (d)
18	Glutamate	2.06 (m), 2.37 (m), 3.76 (m)
19	Glutamine	2.12 (m), 2.44 (m), 3.70 (m)
20	Glycerol	3.54 (dd), 3.62 (dd)
21	Glutathione	2.54 (m); 2.97 (dd)
22	Glycine	3.51 (s)
23	3-hydroxybutyrate	1.20 (d); 2.400 (m); 2.430 (m)
24	Hypoxanthine	8.17 (s), 8.20 (s)
25	IMP	8.53 (s), 8.21 (s)
26	Inosine	3.88 (m), 3.83 (m), 4.28 (m), 4.44 (m), 6.03 (d), 6.12 (d), 8.18 (s), 8.28 (s)
27	Isoleucine	0.92 (t); 0.99 (d); 1.23 (m); 3.64 (d)
28	leucine	0.94 (d), 0.96 (d), 3.72 (m)
29	Lactate	1.32 (d), 4.11 (q)
30	Malate	4.29 (dd)
31	Methionine	2.14 (m), 2.66 (dd), 3.78 (m)
32	Niacinamide	7.60 (m), 8.71 (dd), 8.94 (m)
33	Phenylalanine	7.32 (m), 7.37 (m), 7.42 (m)
34	Valine	0.99 (d), 1.02 (d), 2.28 (m)
35	Phosphocholine	3.20 (s), 4.17 (m)
36	Pyruvate	7.67 (s)
37	Uracil	5.83 (d)

## Data Availability

The data presented in this study is available within the article or supplementary material.

## Supplementary Materials

The following are available online, Figure S1: Stacked ^1^H-NMR spectra of 6 biological replicates of Beef, Donkey meat, Chicken and Chevon, Figure S2: Loading column plot of OPLS-DA separating CM, BM, DM and MS, Figure S3: (A) Score plot (PLS1 vs. PLS3) of OPLS-DA based on whole range of ^1^H-NMR signals (δ 0.5–δ 10.0) of “CM” = Chicken; ‘‘MS’’ = Chevon; ‘‘BM’’ = Beef; “DM” = Donkey (B) Validation through 200 permutation test, Figure S4: Loading column plot of OPLS-DA separating BM, DM and MS. 1, isoleucine; 2, leucine; 3, lactate; 4, Acetate; 5, glutamate; 6, 3-hydroxybutyrate; 7, creatine; 8, phosphocholine; 9, carnitine; 10, glycerol; 11, inosine; 12, malate; 13, uracil; 14, carnosine; 15, hypoxanthine; 16, formate.

## Sample Availability

All the data processed and used during the current study is available from the Institute of Industrial Biotechnology, Government College University Lahore on request.
