# Supplementary material for: 1H-NMR-Based Metabolomics: An Integrated Approach for the Detection of the Adulteration in Chicken, Chevon, Beef and Donkey Meat
Source: Molecules. 2021 Jul 30;26(15):4643. doi: 10.3390/molecules26154643 (PMC8347375; doi:10.3390/molecules26154643)
Supplement: Supplementary file 1 [file molecules-26-04643-s001.zip › Supplementary table.pdf]

Table S1. Relative quantification of metabolites in different meat samples.

Sr. No.	Metabolites	Relative Concentration of Metabolites ± Standard Error Mean				Probability					
		**CM**	**MS**	**BM**	**DM**	**CM × MS**	**CM × BM**	**CM × DM**	**MS × BM**	**MS × DM**	**BM × DM**
**1**	Acetate	0.0307 ± 0.0041	0.0127 ± 0.0022	0.0179 ± 0.0026	0.0164 ± 0.0048	**	ns	*	ns	ns	ns
**2**	Lactate	0.628 ± 0.0481	0.383 ± 0.0824	0.264 ± 0.0582	0.181 ± 0.0364	*	**	***	ns	ns	ns
**3**	Alanine	0.0297 ± 0.0054	0.0107 ± 0.0025	0.0137 ± 0.0025	0.0050 ± 0.0006	***	**	****	ns	ns	ns
**4**	α-mannose	0.0011 ± 0.0020	0.0059 ± 0.0018	0.0033 ± 0.0012	0.0080 ± 0.0014	ns	ns	ns	ns	ns	ns
**5**	Asparagine	0.0066 ± 0.0005	0.0091 ± 0.0020	0.0068 ± 0.0008	0.0115 ± 0.0022	ns	ns	ns	ns	ns	ns
**6**	Biotin	0.0102 ± 0.0011	0.0049 ± 0.0007	0.0032 ± 0.0004	0.0028 ± 0.0004	***	****	****	ns	ns	ns
**7**	Butyric acid	0.0030 ± 0.0009	0.0043 ± 0.0009	0.0032 ± 0.0005	0.0043 ± 0.0007	ns	ns	ns	ns	ns	ns
**8**	Betaine	0.0311 ± 0.0017	0.0365 ± 0.0060	0.0180 ± 0.0034	0.0158 ± 0.0019	ns	ns	*	**	**	ns
**9**	Carnosine	0.0006 ± 0.0003	0.0008 ± 0.0001	0.0006 ± 0.0001	0.0014 ± 0.0002	ns	ns	ns	ns	ns	ns
**10**	Carnitine	0.0495 ± 0.0035	0.167 ± 0.0721	0.339 ± 0.0853	0.176 ± 0.0798	ns	*	ns	ns	ns	ns
**11**	Choline	0.115 ± 0.0121	0.0366 ± 0.0039	0.0360 ± 0.0056	0.0241 ± 0.0051	****	****	****	ns	ns	ns
**12**	Creatine	0.287 ± 0.0213	0.152 ± 0.0315	0.244 ± 0.0175	0.148 ± 0.0325	**	ns	**	ns	ns	ns
**13**	Dimethylamine	0.0037 ± 0.0001	0.0033 ± 0.0005	0.0038 ± 0.0002	0.0014 ± 0.0002	ns	ns	***	ns	**	***
**14**	Formate	0.0007 ± 0.0003	0.0013 ± 0.0003	0.0012 ± 0.0003	0.0061 ± 0.0014	ns	ns	**	ns	**	**
**15**	Glutamine	0.0071 ± 0.0007	0.0182 ± 0.0020	0.0210 ± 0.0033	0.0211 ± 0.0042	ns	**	**	ns	ns	ns
**16**	Glycerol	0.0060 ± 0.0022	0.0063 ± 0.0011	0.0049 ± 0.0012	0.0055 ± 0.0011	ns	ns	ns	ns	ns	ns
**17**	Glutathione	0.0582 ± 0.0064	0.0167 ± 0.0029	0.0151 ± 0.0008	0.009 ± 0.0016	****	****	****	ns	ns	ns
**18**	Hypoxanthine	0.0019 ± 0.0005	0.0113 ± 0.0016	0.0007 ± 0.0003	0.0055 ± 0.0030	**	ns	ns	***	ns	ns
**19**	Inosine	0.0026 ± 0.0008	0.0050 ± 0.0010	0.005 ± 0.0002	0.0010 ± 0.0002	ns	ns	ns	***	***	ns
**20**	Isoleucine	0.0183 ± 0.0046	0.0045 ± 0.0008	0.0004 ± 0.0005	0.0013 ± 0.0002	**	**	***	ns	ns	ns
**21**	Leucine	0.0313 ± 0.0028	0.0176 ± 0.0032	0.0139 ± 0.0011	0.0040 ± 0.0008	**	***	****	ns	**	*
**22**	Malate	0.0179 ± 0.0012	0.0157 ± 0.0042	0.0047 ± 0.006	0.0030 ± 0.0007	ns	**	**	*	**	ns
**23**	Methionine	0.0092 ± 0.0024	0.0083 ± 0.0036	0.0022 ± 0.0004	0.0029 ± 0.0017	ns	ns	ns	ns	ns	ns
**24**	Phenylalanine	0.0043 ± 0.0010	0.0008 ± 0.0002	0.0007 ± 0.0003	0.0005 ± 0.0004	**	**	**	ns	ns	ns
**25**	Valine	0.0113 ± 0.0014	0.0021 ± 0.0007	0.0040 ± 0.0008	0.0009 ± 0.0001	****	***	****	ns	ns	ns
**26**	Pyruvate	0.0174 ± 0.0049	0.0086 ± 0.0019	0.0064 ± 0.0018	0.0044 ± 0.0010	ns	*	*	ns	ns	ns
**27**	Phosphocholine	0.0914 ± 0.0069	0.0350 ± 0.090	0.0991 ± 0.0221	0.0360±	ns	ns	ns	*	ns	*
**28**	Glucose	0.0012 ± 0.0015	0.0190 ± 0.0032	0.0063 ± 0.0023	0.0163 ± 0.0030	ns	ns	**	ns	ns	ns
**29**	Glutamate	0.0107 ± 0.0011	0.0212 ± 0.0051	0.0127 ± 0.0027	0.0165 ± 0.0046	ns	ns	ns	ns	ns	ns
**30**	3-Hydroxybutyrate	0.0181 ± 0.0013	0.0098 ± 0.0008	0.0085 ± 0.0007	0.0032 ± 0.0001	****	****	****	ns	****	***

* indicates *p*-value ($p < 0.05$), ** indicates *p*-value ($p < 0.01$), *** indicates *p*-value ($p < 0.001$), **** indicates *p*-value ($p < 0.0001$), "ns" indicates no significance.
